# Feasibility of asymmetric stretch assessment in the ascending aortic wall with DENSE cardiovascular magnetic resonance

**DOI:** 10.1186/1532-429X-16-6

**Published:** 2014-01-09

**Authors:** Henrik Haraldsson, Michael Hope, Gabriel Acevedo-Bolton, Elaine Tseng, Xiaodong Zhong, Frederick H Epstein, Liang Ge, David Saloner

**Affiliations:** 1 Department of Radiology and Biomedical Imaging, University of California, San Francisco, CA, USA; 2 Veterans Affairs Medical Center, San Francisco, CA, USA; 3 Department of Surgery, University of California, San Francisco, CA, USA; 4 MR R&D Collaborations, Siemens Healthcare, Atlanta, GA, USA; 5 Department of Biomedical Engineering, University of Virginia, Charlotteville, VA, USA; 6 VAMC/UCSF, Radiology 114-D, Bldg 203, Rm BA-51, 4150 Clement Street, San Francisco, CA 94530, USA

**Keywords:** Aorta, DENSE, Stretch, Stiffness, Bicuspid aortic valves, Cardiovascular magnetic resonance

## Abstract

**Background:**

Vessel diameter is the principal imaging parameter assessed clinically for aortic disease, but adverse events can occur at normal diameters. Aortic stiffness has been studied as an additional imaging-based risk factor, and has been shown to be an independent predictor of cardiovascular morbidity and all-cause mortality. Reports suggest that some aortic pathology is asymmetric around the vessel circumference, a feature which would not be identified with current imaging approaches. We propose that this asymmetry may be revealed using Displacement Encoding with Stimulated Echoes (DENSE). The objective of this study is to investigate the feasibility of assessing asymmetric stretch in healthy and diseased ascending aortas using DENSE.

**Methods:**

Aortic wall displacement was assessed with DENSE cardiovascular magnetic resonance (CMR) in 5 volunteers and 15 consecutive patients. Analysis was performed in a cross-sectional plane through the ascending aorta at the pulmonary artery. Displacement data was used to determine the wall stretch between the expanded and resting states of the aorta, in four quadrants around the aortic circumference.

**Results:**

Analysis of variance (ANOVA) did not only show significant differences in stretch between groups of volunteers (p < 0.001), but also significant differences in stretch along the circumference of the aorta (p < 0.001), indicating an asymmetric stretch pattern. Furthermore, there is a significant difference in the asymmetry between volunteers and different groups of patients (p < 0.01).

**Conclusions:**

Evaluation of asymmetric stretch is feasible in the ascending aorta with DENSE CMR. Clear differences in stretch are seen between patients and volunteers, with asymmetric patterns demonstrated around the aortic circumference.

## Background

Imaging plays a central role in the diagnosis and management of patients with aortic disease. Currently, vessel diameter is the principal imaging parameter used in clinical assessments [1]. Other imaging based measurements, including unique geometries [2-4] and wall stress [5,6], have been proposed for further risk-stratification, predominantly for the abdominal and the descending thoracic aorta, but have not yet become part of clinical routine.

Aortic stiffness is a promising measure that has shown importance as an independent predictor of cardiovascular morbidity and all-cause mortality [7]. It is a broad and early manifestation of pathologic changes within the vessel wall that can be measured with imaging using pulse wave velocity (PWV) or aortic distensibility. For example, patients with Marfan syndrome, a genetic disorder of the connective tissue, but normal-sized aortas present with abnormal aortic distensibility and stiffness indices as demonstrated by cardiovascular magnetic resonance (CMR) [8,9]. Both PWV and aortic distensibility provide an integrated measure of the aortic stiffness: PWV assesses overall stiffness along a vascular segment, and aortic distensibility provides an average stiffness around the aortic circumference. However, in some clinical contexts, the progression of aortic disease has been shown to be asymmetric. Asymmetric dilation of the ascending aorta has been linked to aortic valve disease [10,11], and the age-related widening of the aortic arch is noted to be greater anteriorly than posteriorly [12]. Asymmetry is also seen in matrix alterations with congenital valve disease, where smooth muscle cell apoptosis is more pronounced in the outer curvature of the ascending aorta [13]. These asymmetric findings may be related to the eccentric flow that has been demonstrated in patients with bicuspid aortic valve (BAV) [14,15]. Ex vivo experiments have also shown asymmetric mechanical properties in the aortic wall [16,17].

A technique that is able to resolve regional variations in stiffness around the aortic circumference in vivo would be required for assessment of these asymmetric pathologies. One such technique is displacement encoding with stimulated echoes (DENSE) [18], which has been primarily used to study myocardial mechanics. This technique uses the phase of the CMR signal to acquire the in- and through-plane displacement between the time of a pre-pulse and the time of the readout. Since it does not use magnitude data to track motion, it is less dependent on image resolution than techniques that resolve tag lines or anatomical features. The displacement maps obtained from DENSE are used to calculate stretch on a regional basis within the tissue. Applying DENSE to the ascending aorta is challenging because the ascending aortic wall is relatively thin, of the order of 2–3 mm [19,20], while still subject to the complex motion caused by the heart and by breathing. The objective of this study is to investigate the feasibility of assessing asymmetric wall stretch along the circumference of the healthy and diseased ascending aorta using DENSE.

## Methods

Fifteen consecutive patients with congenital valve disease or dilated aortas, who were referred by cardiothoracic surgery for evaluation of their ascending aortas, were included in the study along with five healthy volunteers. The study was approved by the Committee on Human Research at the University of California San Francisco Medical Center and the Institutional Review Board of the San Francisco VA Medical Center. Informed consent was obtained from all subjects.

Patients were grouped according to their clinical patient history. Ascending aortic diameter measurement were made at standard levels [1,21] based on their most recent computed tomography angiograms.

### Data acquisition

**Figure 1:**
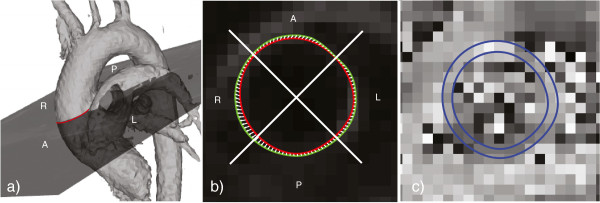
**DENSE in the tubular portion of the ascending aorta. a)** Cine and DENSE images were acquired in a slice through the tubular portion of the ascending aorta at the pulmonary artery. **b)** The circumference of the ascending aorta at diastole was delineated (red line) and the displacement field (white arrows) was used to assess its relationship with the systolic circumference (green line). Stretch analysis was subdivided into anterior (A), right (R), posterior (P), and left (L) quadrants. **c)** phase of the DENSE data that correspond to displacement in the anterior-posterior direction. The blue lines illustrate the inner and outer boarder of the vessel wall.

CMR data was acquired on Siemens Avanto and Philips Achieva systems at 1.5 T. The acquisition started with CINE imaging of a slice through the tubular portion of the aorta, proximal to the bifurcation of the pulmonary artery (Figure 1). That data was used to find the time point of maximum expansion, defined as maximum lumen area. The CINE imaging was followed by a DENSE acquisition starting with its initial position encoding at the time point of maximum expansion, and with a single time point readout performed in diastole. DENSE data was acquired with an ECG-triggered, navigator-gated acquisition using balanced 4-point displacement encoding [22] with 3- to 6- point phase cycling [23] with in- and through-plane encoding frequencies of 0.08-0.11 cycles/mm. The sequence performed segmented spiral acquisition with the following parameters: TE of 1.14 ms, TR of 18–27 ms, 8 spiral interleaves per image, 1 spiral interleave per heart beat, FOV of 360 × 360 to 410 × 410 mm^2^, slice thickness of 8 mm, base resolution 148 × 148 to 180 × 180, resulting in a reconstructed pixel size of 2.3 × 2.3 to 2.4 × 2.4 mm^2^. CINE steady state free precession (SSFP) was acquired with a TE of 1.27-1.72 ms, TR of 3–3.4 ms, resulting in an actual temporal resolution of 42–75 ms, and a pixel size of 1.4-1.9 mm. An acceleration factor of 2 was used for the CINE SSFP acquisition using GRAPPA (Siemens Avanto) or SENSE (Philips Achieva) [24,25].

### Post processing

The post processing of the DENSE data was implemented in MATLAB (MathWorks Inc., Natick, Massachusetts). The processing included five steps

1) **Segmentation and delineation of the aortic circumference:** The inner and outer border of the vessel was segmented in the DENSE magnitude image using splines. Data points outside the segmented aorta were not considered for further analysis. Individual pixels within the segmentation with low magnitude, and thereby unreliable phase, were also excluded in this step. The middle of the aortic circumference was delineated using a separate spline.

2) **Phase unwrapping of the DENSE data:** Normalized averaging [26] was used to automatically weigh the reliability of the pixels – pixels with larger signal magnitude have more reliable phase data. Normalized averaging was performed with a certainty related to the magnitude of the CMR signal and a Gaussian applicability standard deviation of 1 pixel width. A Poisson solver [27] was used to unwrap the phase data [28].

3) **Tracking:** 100 points were automatically distributed equidistantly along the delineation of the diastolic aortic circumference. The displacement field obtained by DENSE was used to find the corresponding location of the points during systole. These systolic points where matched to a periodic spline with 5 pieces to reduce the influence of noise.

4) **Calculate stretch:** Stretch was calculated by comparing the change in distance between adjacent points along the expanded and resting state splines.

5) **Quadrant analysis:** To present regional stretch along the circumference, the aorta was divided into four quadrants (Figure 1): anterior (A); right (R); posterior (P); and left (L).

The relative stretch difference for each quadrant was calculated to study the asymmetry of the stretch within the groups. The relative stretch difference was defined as (S_quadrant_ - S_overall_)/S_overall_ where S_overall_ is the average stretch along the whole circumference and S_quadrant_ is the average stretch within the quadrant.

### Relative cross-sectional area change from CINE SSFP

A cross-sectional area of the aorta was obtained from the CINE SSFP slice using the segmentation software *Segment* (http://segment.heiberg.se) [29]. The relative cross-sectional area changes were calculated based on the maximum, A_max_, and the minimum, A_min_, cross-sectional area, and was defined as (A_max_ - A_min_)/A_min_.

### Statistical analysis

The stretch was analyzed using two-way analysis of variance (ANOVA) and Tukey’s range test was used to compare groups and quadrants. The correlation of the average stretch along the whole circumference obtained by DENSE, and the relative cross-sectional area change obtained by CINE imaging, was compared using Pearson’s correlation coefficient.

## Results

Three of the patients and one of the volunteers were excluded because of poor image quality caused by motion artifact. Of the remaining twelve patients, eight had dilated aortas and the remaining four had a BAV with non-dilated aortas. A summary of the groups is provided in Table 1.

**Table 1:** Subject groups

**Group**	**Volunteers**	**Dilated patients**	**Non-dilated BAV**
N	4	8	4
Age [years]	30 ± 5	64 ± 5	43 ± 6
Systolic blood pressure [mmHg]	109 ± 13	124 ± 12	132 ± 14
Diastolic blood pressure [mmHg]	65 ± 5	79 ± 13	70 ± 10
Pulse pressure [mmHg]	44 ± 13	45 ± 5	62 ± 5
Relative cross-sectional area Change [%]	40 ± 6	7 ± 3	24 ± 11

The ANOVA test showed a significant difference (p < 0.001) between groups; healthy volunteers, patients with non-dilated aortas and BAV, and patients with dilated aortas. The test also showed a significant difference between the quadrants (p < 0.001). Furthermore, there was a significant difference between the quadrants for the different groups (p < 0.01).

**Figure 2:**
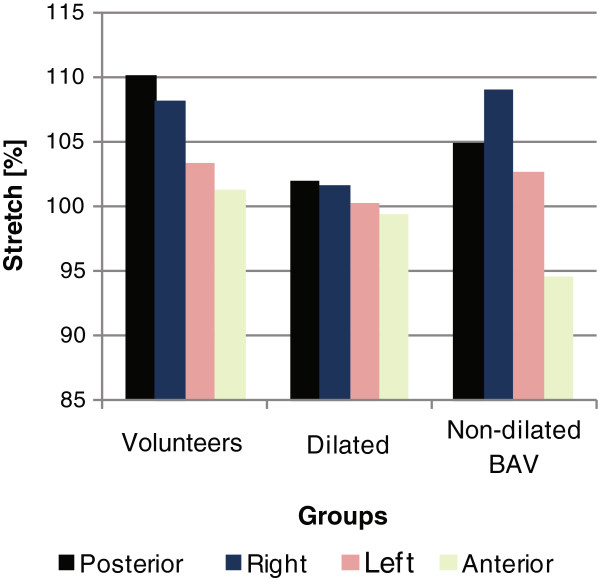
**Stretch by quadrant for volunteers and patient groups.** The overall stretch was 106% for the volunteers, 103% for the non-dilated BAV patients, and 101% for the dilated patient group. Patients with dilated aorta had a decreased overall stretch compared to non-dilated aortas. The volunteers show the greatest difference in stretch between the posterior and anterior quadrants (p < 0.05) while the non-dilated BAV patients had the greatest stretch in the right quadrant and the least stretch in the anterior quadrant (p < 0.001).

The greatest overall stretch (106%) was seen in the volunteers (Figure 2). Within the patient group, those with non-dilated aortas and BAV had greater overall stretch (103%) than did patients with dilated aortas (101%).

**Figure 3:**
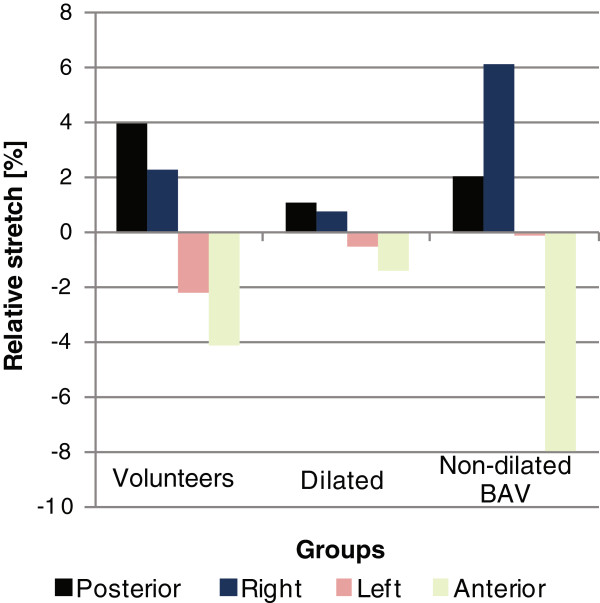
**The relative stretch in the different quadrants in relation to the overall stretch.** Both the volunteers and patients with non-dilated aortas and BAV demonstrated more asymmetric stretch around the aortic circumference. The volunteers show the greatest difference between the posterior and anterior. The non-dilated BAV, on the other hand, showed the greatest difference between the right and anterior.

**Figure 4:**
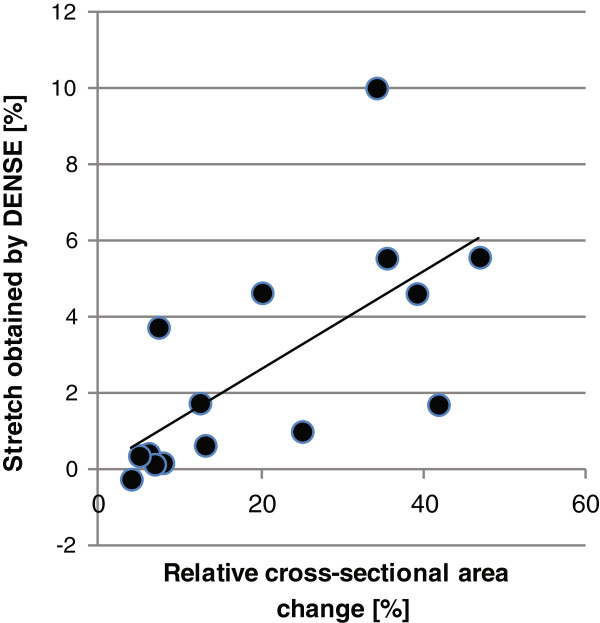
**Stretch and cross-sectional area change.** Stretch obtained with DENSE showed good linear correlation (r = 0.68) with the relative cross-sectional area change.

Both the volunteers and the patients with BAV and non-dilated aortas demonstrated more asymmetric stretch around the aortic circumference than patients with dilated aortas (Figure 3). The volunteers showed the greatest difference in stretch between the posterior and anterior quadrants (p < 0.05). The non-dilated BAV patients showed a different asymmetric stretch pattern than the volunteers. They had the greatest stretch in the right quadrant and the least stretch in the anterior quadrant (p < 0.001). The stretch obtained with DENSE showed good linear correlation (r = 0.68) with the relative cross-sectional area change (Figure 4).

## Discussion

We have demonstrated that evaluation of asymmetric stretch in the ascending aorta is feasible with DENSE CMR. Our data indicates that an asymmetric stretch pattern exists in the aorta, and that this asymmetry differs between healthy volunteers, patients with BAV and non-dilated aortas, and patients with dilated aortas.

Previous methods to make in vivo measurements of the stretch in the aorta have been limited to the overall stretch. This overall stretch can be used to measure the stiffness via aortic distensibility; the relation between the relative change in area and the central pulse pressure. The method presented in this work furthers to this idea by resolving the stretch along the circumference of the aorta, and brings us closer to resolving the stiffness along the circumference. It is however important to realize that both the hemodynamic forces and the mechanical properties in the wall are asymmetric and that it is the contribution of the two that result in the stretch. Previous studies have reported significant differences in the material properties along the circumference of the aorta [16,17]. It is interesting to note that the right quadrant, having a positive relative stretch in our study (Figure 3) and that is likely to be affected by greater hemodynamic forces, was reported to commonly be the stiffest quadrant at both at low and high elastic stress [16].

Applying DENSE to the ascending aorta is challenging due to the relatively thin aortic wall. The aortic wall is much thinner than the wall of the left ventricle which is commonly studied with this technique. It is intuitive to think that a corresponding increase in spatial resolution may be required. Although high spatial resolution is always desirable; DENSE uses the phase of the data to acquire the displacement, and this affects the choice of resolution in two ways. First: in DENSE the phase of a single voxel is directly proportional to its displacement. As long as the voxel of interest is located in the vessel wall, increased resolution would not improve the ability to track motion in the same way as techniques that rely on the ability to resolve tag lines or anatomical features in the tissue of interest. Second, the fact that DENSE uses the phase of the signal affects the tradeoff between the spatial resolution and the signal-to-noise (SNR). Increased resolution results in decreased SNR due to the decreased voxel size. The decreased SNR increases the standard deviation of the phase, and consequently decreases the accuracy of the displacement acquired. So contrary to more image processing based techniques, the accuracy of the motion obtained with DENSE decreases if the resolution becomes too high. Taking these two points into account, the resolution was chosen to be able to distinguish the middle of the wall with sufficient SNR, rather than trying to increase the resolution at the cost of SNR. A potential drawback of a lower resolution is the partial volume effect, where MR signal from both wall and blood contributes to signal from a single voxel. However, DENSE has an intrinsic black blood effect, because the blood that was displacement encoded at the initial timing moves out the imaging plane and does not contribute to the signal when performing the readout. Therefore, the phase of the net magnetization vector in a voxel is dominated by that of the tissue and is relatively insensitive to contributions from blood. DENSE has been used in vessel wall applications before, where the in-plane displacement was used to acquire strain in the common carotid artery [30]. Unlike the common carotid arteries, the ascending aorta lacks the soft surrounding tissue that deforms with the vessel wall and contributes to the CMR signal. This emphasizes the importance of obtaining high signal from the aortic vessel wall itself. The ascending aorta is also less pipe-like and is therefore more likely to deform in a more complex manner, which necessitates acquisition of both in- and through-plane displacement for full characterization. For the application in this work, we acquired in- and though-plane DENSE data at a single time frame to increase SNR over that which can be obtained from conventional multi-phase cine acquisition [31]. We also chose to apply the position encoding prepulse at the time point of maximum aortic cross-sectional area and the readout in late diastole. This resembles the strategy previously used to study mechanical properties of the heart [32]. Our experience is that this approach resulted in better image quality for the ascending aorta compared to the conventional timing, where the initial position encoding is performed at the R-wave. Due to the thin aortic wall, we speculate that it is important to acquire the data during the phase of least motion and reduced variability between heartbeats.

Compared to conventional stiffness analysis based on cross-sectional changes in aortic area, data derived from DENSE exhibited a good linear correlation (r = 0.68). The lack of a stronger correlation may be related to the fact that DENSE acquisition in this study was encoded in all three directions. Analysis with DENSE takes into account all three dimensions, and thereby through-plane motion, which is not accounted for in cross-sectional analysis. Additionally, our DENSE sequence does not acquire the maximum change as does the relative cross-sectional area change, but rather the displacement between two predefined time points.

**Figure 5:**
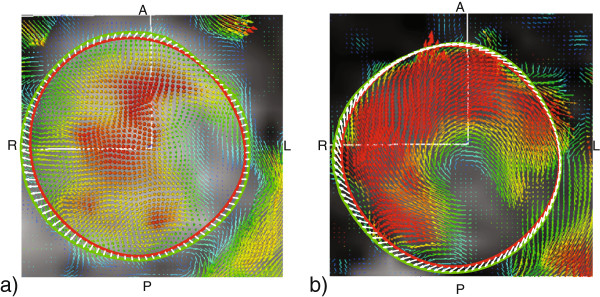
**Comparison of flow and aortic stretch in a volunteer and BAV patient.** The volunteer **(a)** has a relatively parabolic flow profile with faster flow in the center of the aorta (red arrows), and slower flowing blood closer to the vessel wall (green/blue arrows). The displacement of the vessel wall (white arrows) shows the stretch of the aorta wall. A patient with non-dilated aorta and BAV **(b)** exhibits more eccentric flow, with faster and helical flow peripherally at the vessel wall. The corresponding displacement field indicates that the vessels wall both stretches and rotates corresponding to the circular flow, which is much more pronounced than in the volunteer data.

Our data shows asymmetric stretch in the non-dilated aorta. An asymmetric stretch is expected with normal anatomy based on the typical offset of axes between the left ventricle and aortic root [33]. This may be more pronounced when eccentric blood flow is present as has been shown with BAV [14,15]. From a 3D time resolved flow acquisition included in our protocol, but not part of our suggested methodology, volunteers demonstrate a relatively parabolic flow compared to patients with BAV who have eccentric systolic flow (exemplified in Figure 5). The displacement field of this patient with BAV indicates that the vessel not only expands, but also rotates corresponding to the circular flow, which is not seen in the volunteer data. Such findings may help explain the asymmetric dilatation that has been reported in these patients with BAV [10,11].

Strain was successfully acquired in 16 out of 20 volunteers, corresponding to an 80% success rate. Four of the volunteers were excluded due to insufficient image quality, most likely caused by motion artifacts. Even though it is challenging to identify the exact cause of the motion artifacts for each individual case, it is worth discussing two potential sources. The first is major movement by the patient, e.g. when a patient readjusts their position due to discomfort. This source of motion may always be more cumbersome for lengthier data acquisitions. The data acquired in this study was part of a lengthy comprehensive examination of the ascending aorta, and the DENSE acquisition was performed in the end of this examination. A shorter examination time would most likely have increased the patient compliance. The second source of motion artifacts is an improper timing of the readout. As the aortic wall is a thin structure with a cyclic motion, it is important that data is acquired in the diastolic plateau phase which is more reproducible. Errors in the timing of this phase would produce more variability between the heart beats, and consequently more motion artifacts. On-line post processing of the DENSE data would make it easier to determine if the data need to be reacquired while the patient is in the scanner.

The objective of the study was to demonstrate the feasibility of using DENSE to assess stretch in the ascending aorta. Hence, the volunteers were not age matched, but rather groups with different challenging features were used to evaluate the technique. The volunteers included, 1) young healthy volunteers with compliant aortas and greater motion of the aorta, 2) BAV patients known for their asymmetric disease, and 3) patients with dilated stiffer aortas. The study shows that it is feasible to use DENSE to assess stretch along the circumference of the ascending aorta, and the results show that the stretch is asymmetric. The difference between groups, the asymmetry of stretch along the circumference, and the difference in asymmetry between the groups shows that the proposed method is feasible, and that unique stretch patterns along the circumference can be found. Future works include matched groups needed to identify independent relationships.

## Conclusion

We have shown that DENSE CMR is feasible for evaluation of asymmetric stretch around the circumference of the ascending aorta. Significant differences in stretch are seen between patients and volunteers, and between quadrants along the circumference of the aorta, i.e. asymmetric stretch. Significant differences in the asymmetry were also seen between groups. With its ability to resolve regional stretch differences around the aortic circumference, we believe that DENSE is an important tool for assessment of pathology in the aorta.

## Competing interests

The authors declare that they have no competing interests.

## Authors’ contributions

HH participated in the study design, data acquisition, data analysis, and coordinated and drafted the manuscript. MH participated in study design, patient recruitment, data analysis, and helped draft the manuscript. GAB assisted with data acquisition and analysis, and helped draft the manuscript. ET participated in study design and coordinated patient recruitment. XZ and FE participated in data acquisition methodology. LG participated in study design. DS participated in study design, data analysis, and helped draft the manuscript. All authors read and approved the final manuscript.

## References

[B1] Hiratzka LF Bakris GL Beckman JA Bersin RM Carr VF Casey DE Jr Eagle KA Hermann LK Isselbacher EM Kazerooni EA Kouchoukos NT Lytle BW Milewicz DM Reich DL Sen S Shinn JA Svensson LG Williams DM ACCF/AHA/AATS/ACR/ASA/SCA/SCAI/SIR/STS/SVM guidelines for the diagnosis and management of patients with Thoracic Aortic Disease: a report of the American College of Cardiology Foundation/American Heart Association Task Force on Practice Guidelines, American Association for Thoracic Surgery, American College of Radiology, American Stroke Association, Society of Cardiovascular Anesthesiologists, Society for Cardiovascular Angiography and Interventions, Society of Interventional Radiology, Society of Thoracic Surgeons, and Society for Vascular Medicine Circulation 2010 2010 121 e266 e369 20233780 10.1161/CIR.0b013e3181d4739e

[B2] Poullis MP Warwick R Oo A Poole RJ Ascending aortic curvature as an independent risk factor for type A dissection, and ascending aortic aneurysm formation: a mathematical model Eur J Cardiothorac Surg 2008 33 995 1001 10.1016/j.ejcts.2008.02.029 18434179

[B3] Goergen CJ Azuma J Barr KN Magdefessel L Kallop DY Gogineni A Grewall A Weimer RM Connolly AJ Dalman RL Taylor CA Tsao PS Greve JM Influences of aortic motion and curvature on vessel expansion in murine experimental aneurysms Arterioscler Thromb Vasc Biol 2011 31 270 279 10.1161/ATVBAHA.110.216481 21071686 PMC3024449

[B4] Doyle BJ Callanan A Burke PE Grace PA Walsh MT Vorp DA McGloughlin TM Vessel asymmetry as an additional diagnostic tool in the assessment of abdominal aortic aneurysms J Vasc Surg 2009 49 443 454 10.1016/j.jvs.2008.08.064 19028061 PMC2666821

[B5] Fillinger MF Marra SP Raghavan ML Kennedy FE Prediction of rupture risk in abdominal aortic aneurysm during observation: wall stress versus diameter J Vasc Surg 2003 37 724 732 10.1067/mva.2003.213 12663969

[B6] Shang EK Nathan DP Sprinkle SR Vigmostad SC Fairman RM Bavaria JE Gorman RC Gorman JH 3rd Chandran KB Jackson BM Peak wall stress predicts expansion rate in descending thoracic aortic aneurysms Ann Thorac Surg 2013 95 593 598 10.1016/j.athoracsur.2012.10.025 23245445 PMC4037886

[B7] Cavalcante JL Lima JAC Redheuil A Al-Mallah MH Aortic stiffness: current understanding and future directions J Am Coll Cardiol 2011 57 1511 1522 10.1016/j.jacc.2010.12.017 21453829

[B8] Adams JN Brooks M Redpath TW Smith FW Dean J Gray J Walton S Trent RJ Aortic distensibility and stiffness index measured by magnetic resonance imaging in patients with Marfan’s syndrome Br Heart J 1995 73 265 269 10.1136/hrt.73.3.265 7727188 PMC483810

[B9] Groenink M de Roos A Mulder BJ Verbeeten B Jr Timmermans J Zwinderman AH Spaan JA van der Wall EE Biophysical properties of the normal-sized aorta in patients with Marfan syndrome: evaluation with MR flow mapping Radiology 2001 219 535 540 10.1148/radiology.219.2.r01ma01535 11323484

[B10] Cotrufo M Della Corte A The association of bicuspid aortic valve disease with asymmetric dilatation of the tubular ascending aorta: identification of a definite syndrome J Cardiovasc Med (Hagerstown) 2009 10 291 297 10.2459/JCM.0b013e3283217e29 19242284

[B11] Lu MT Thadani SR Hope MD Quantitative assessment of asymmetric aortic dilation with valve-related aortic disease Acad Radiol 2013 20 10 15 10.1016/j.acra.2012.07.012 22951111

[B12] Redheuil A Yu W-C Mousseaux E Harouni AA Kachenoura N Wu CO Bluemke D Lima JAC Age-related changes in aortic arch geometry: relationship with proximal aortic function and left ventricular mass and remodeling J Am Coll Cardiol 2011 58 1262 1270 10.1016/j.jacc.2011.06.012 21903061 PMC3508703

[B13] Della Corte A Quarto C Bancone C Castaldo C Di Meglio F Nurzynska D De Santo LS De Feo M Scardone M Montagnani S Cotrufo M Spatiotemporal patterns of smooth muscle cell changes in ascending aortic dilatation with bicuspid and tricuspid aortic valve stenosis: focus on cell-matrix signaling J Thorac Cardiovasc Surg 2008 135 8 18 18.e1–2 10.1016/j.jtcvs.2007.09.009 18179910

[B14] Viscardi F Vergara C Antiga L Merelli S Veneziani A Puppini G Faggian G Mazzucco A Luciani GB Comparative finite element model analysis of ascending aortic flow in bicuspid and tricuspid aortic valve Artif Organs 2010 34 1114 1120 10.1111/j.1525-1594.2009.00989.x 20618222

[B15] Hope MD Hope TA Meadows AK Ordovas KG Urbania TH Alley MT Higgins CB Bicuspid aortic valve: four-dimensional MR evaluation of ascending aortic systolic flow patterns Radiology 2010 255 53 61 10.1148/radiol.09091437 20308444

[B16] Choudhury N Bouchot O Rouleau L Tremblay D Cartier R Butany J Mongrain R Leask RL Local mechanical and structural properties of healthy and diseased human ascending aorta tissue Cardiovasc Pathol 2009 18 83 91 10.1016/j.carpath.2008.01.001 18402840

[B17] Iliopoulos DC Deveja RP Kritharis EP Perrea D Sionis GD Toutouzas K Stefanadis C Sokolis DP Regional and directional variations in the mechanical properties of ascending thoracic aortic aneurysms Med Eng Phys 2009 31 1 9 10.1016/j.medengphy.2008.03.002 18434231

[B18] Aletras AH Ding S Balaban RS Wen H DENSE: displacement encoding with stimulated echoes in cardiac functional MRI J Magn Reson 1999 137 247 252 10.1006/jmre.1998.1676 10053155 PMC2887318

[B19] Li AE Kamel I Rando F Anderson M Kumbasar B Lima JAC Bluemke DA Using MRI to assess aortic wall thickness in the multiethnic study of atherosclerosis: distribution by race, sex, and age AJR Am J Roentgenol 2004 182 593 597 10.2214/ajr.182.3.1820593 14975953

[B20] Azadani AN Chitsaz S Mannion A Mookhoek A Wisneski A Guccione JM Hope MD Ge L Tseng EE Biomechanical properties of human ascending thoracic aortic aneurysms Ann Thorac Surg 2013 96 50 58 10.1016/j.athoracsur.2013.03.094 23731613

[B21] Mendoza DD Kochar M Devereux RB Basson CT Min JK Holmes K Dietz HC Milewicz DM LeMaire SA Pyeritz RE Bavaria JE Maslen CL Song H Kroner BL Eagle KA Weinsaft JW GenTAC (National Registry of Genetically Triggered Thoracic Aortic Aneurysms and Cardiovascular Conditions) Study Investigators Impact of image analysis methodology on diagnostic and surgical classification of patients with thoracic aortic aneurysms Ann Thorac Surg 2011 92 904 912 10.1016/j.athoracsur.2011.03.130 21723533

[B22] Zhong X Helm PA Epstein FH Balanced multipoint displacement encoding for DENSE MRI Magn Reson Med 2009 61 981 988 10.1002/mrm.21851 19189288 PMC2772058

[B23] Zhong X Spottiswoode BS Meyer CH Kramer CM Epstein FH Imaging three-dimensional myocardial mechanics using navigator-gated volumetric spiral cine DENSE MRI Magn Reson Med 2010 64 1089 1097 10.1002/mrm.22503 20574967 PMC2946451

[B24] Griswold MA Jakob PM Heidemann RM Nittka M Jellus V Wang J Kiefer B Haase A Generalized autocalibrating partially parallel acquisitions (GRAPPA) Magn Reson Med 2002 47 1202 1210 10.1002/mrm.10171 12111967

[B25] Pruessmann KP Weiger M Scheidegger MB Boesiger P SENSE: sensitivity encoding for fast MRI Magn Reson Med 1999 42 952 962 10.1002/(SICI)1522-2594(199911)42:5<952::AID-MRM16>3.0.CO;2-S 10542355

[B26] Farnebäck G Polynomial Expansion for Orientation and Motion Estimation Ph.D. thesis 2002 Sweden: Linköping University SE-581 83 Linköping, Sweden, 2002. Dissertation No 790, ISBN, 91-7373-475-6

[B27] Ebbers T Farnebäck G Improving computation of cardiovascular relative pressure fields from velocity MRI J Magn Reson Imaging 2009 30 54 61 10.1002/jmri.21775 19557846

[B28] Moon-Ho Song S Napel S Pelc NJ Glover GH Phase unwrapping of MR phase images using Poisson equation IEEE Trans Image Process 1995 4 667 676 10.1109/83.382500 18290015

[B29] Heiberg E Sjögren J Ugander M Carlsson M Engblom H Arheden H Design and validation of Segment–freely available software for cardiovascular image analysis BMC Med Imaging 2010 10 1 10.1186/1471-2342-10-1 20064248 PMC2822815

[B30] Lin AP Bennett E Wisk LE Gharib M Fraser SE Wen H Circumferential strain in the wall of the common carotid artery: comparing displacement-encoded and cine MRI in volunteers Magn Reson Med 2008 60 8 13 10.1002/mrm.21621 18581403 PMC2886512

[B31] Sigfridsson A Haraldsson H Ebbers T Knutsson H Sakuma H In vivo SNR in DENSE MRI; temporal and regional effects of field strength, receiver coil sensitivity and flip angle strategies Magn Reson Imaging 2011 29 202 208 10.1016/j.mri.2010.08.016 21129876

[B32] Wen H Bennett E Epstein N Plehn J Magnetic resonance imaging assessment of myocardial elastic modulus and viscosity using displacement imaging and phase-contrast velocity mapping Magn Reson Med 2005 54 538 548 10.1002/mrm.20589 16086299 PMC2886520

[B33] Laas J Kleine P Hasenkam MJ Nygaard H Orientation of tilting disc and bileaflet aortic valve substitutes for optimal hemodynamics Ann Thorac Surg 1999 68 1096 1099 10.1016/S0003-4975(99)00780-8 10510028

